# Increases in CSF dopamine in HIV patients are due to the dopamine transporter 10/10-repeat allele which is more frequent in HIV-infected individuals

**DOI:** 10.1007/s00702-013-1086-x

**Published:** 2013-09-06

**Authors:** Anne Horn, Carsten Scheller, Stefan du Plessis, Gabriele Arendt, Thorsten Nolting, John Joska, Sieghart Sopper, Matthias Maschke, Mark Obermann, Ingo W. Husstedt, Johannes Hain, Tongai Maponga, Peter Riederer, Eleni Koutsilieri, the German Competence Network HIV/AIDS

**Affiliations:** 1 Institute of Virology and Immunobiology, University of Würzburg, Versbacher Str. 7, 97078 Würzburg, Germany; 2 Department of Psychiatry, Stellenbosch University, Stellenbosch, South Africa; 3 Department of Neurology, University Hospital of Düsseldorf, Düsseldorf, Germany; 4 Department of Psychiatry, University of Cape Town, Cape Town, South Africa; 5 IFTZ, Medical University Innsbruck, Innsbruck, Austria; 6 Department of Neurology, University Hospital of Duisburg-Essen, Essen, Germany; 7 Department of Neurology, University Hospital of Münster, Münster, Germany; 8 Institute of Mathematics and Informatics, Chair of Mathematics VIII (Statistics), University of Würzburg, Würzburg, Germany; 9 Department of Virology, Stellenbosch University, Stellenbosch, South Africa; 10 Department of Psychiatry, Psychosomatics and Psychotherapy, University of Würzburg, Würzburg, Germany

**Keywords:** Dopamine, DAT, Polymorphism, HIV, CSF, HAND

## Abstract

Dysfunction of dopaminergic neurotransmission has been implicated in HIV infection. We showed previously increased dopamine (DA) levels in CSF of therapy-naïve HIV patients and an inverse correlation between CSF DA and CD4 counts in the periphery, suggesting adverse effects of high levels of DA on HIV infection. In the current study including a total of 167 HIV-positive and negative donors from Germany and South Africa (SA), we investigated the mechanistic background for the increase of CSF DA in HIV individuals. Interestingly, we found that the DAT 10/10-repeat allele is present more frequently within HIV individuals than in uninfected subjects. Logistic regression analysis adjusted for gender and ethnicity showed an odds ratio for HIV infection in DAT 10/10 allele carriers of 3.93 (95 % CI 1.72–8.96; *p* = 0.001, Fishers exact test). 42.6 % HIV-infected patients harbored the DAT 10/10 allele compared to only 10.5 % uninfected DAT 10/10 carriers in SA (odds ratio 6.31), whereas 68.1 versus 40.9 %, respectively, in Germany (odds ratio 3.08). Subjects homozygous for the 10-repeat allele had higher amounts of CSF DA and reduced DAT mRNA expression but similar disease severity compared with those carrying other DAT genotypes. These intriguing and novel findings show the mutual interaction between DA and HIV, suggesting caution in the interpretation of CNS DA alterations in HIV infection solely as a secondary phenomenon to the virus and open the door for larger studies investigating consequences of the DAT functional polymorphism on HIV epidemiology and progression of disease.

## Introduction

HIV infection causes neurological and psychiatric complications manifesting in HIV-associated neurocognitive disorders (HAND) despite antiretroviral therapy (Mothobi and Brew 2012). They occur in various forms and affect medication adherence and quality of life. Research focuses on host factors that may be implicated in HAND pathogenesis, the etiology of HAND; however, remains so far unclear.

Disorganization of dopaminergic circuits in HIV infection has been implicated in the neuropathophysiology of HIV infection, and has been described extensively in the reviews of (Koutsilieri et al. 2002; Purohit et al. 2011). HIV as well as the simian immunodeficiency virus (SIV) causes apparent reduction in postmortem brain dopamine (DA) levels (Kumar et al. 2009; Sardar et al. 1996; Scheller et al. 2005), suggesting that intracellular DA levels are depleted. An explanation for this may be the dysfunction of the DA transporter (DAT) known to regulate DA availability through DA uptake and release. Indeed, decreased DAT has been found in brains of HIV-associated dementia patients with positron emission tomography (PET) (Wang et al. 2004), and HIV proteins have been shown to impair DAT in animals (Maragos et al. 2002). In contrast, increased DAT protein has been detected in striatal specimens of subjects with HIV encephalitis (Gelman et al. 2006) and no alterations of DAT were found with single-photon emission computed tomography (SPECT) in brains of HIV/treatment-naive patients (Scheller et al. 2010). However, DA levels were increased in CSF of HIV/treatment-naive patients compared with uninfected subjects (Scheller et al. 2010), suggesting an altered regulation of extracellular concentrations of DA.

In the current study, we thought that the increase of CSF DA may have been due to the genetic constitution of the subjects and may not have been a direct causal effect of the virus. We specifically hypothesized that humans carrying a specific allele of the DAT functional genetic polymorphism associated with higher availability of DA would (a) have higher levels of CSF DA (b) be present more frequently within HIV individuals and (c) show adverse development of HIV infection compared to those carrying alleles associated with reduced DA availability. To assess our hypothesis we analyzed CSF DA, DAT mRNA expression, determined DAT genetic polymorphisms in uninfected and HIV-infected subjects and investigated whether different ethnicities would influence our assumption. Progression of disease in the infected groups was based on routine virological and immunological measures as well as scores in the HIV dementia scale (HDS), international HIV dementia scale (IHDS) and global deficit score (GDS). Detailed neuropsychological testing has been assessed but will be reported elsewhere.

## Materials and methods

### Study population

A total of 167 people participated in this study: 72 HIV-infected patients and 22 age-matched controls from Germany as well as 54 HIV-infected patients and 19 controls from South Africa (SA). All Germans were white. From SA 88 % were Xhosa (black) and 12 % were colored of mixed racial ancestry. The study was conducted according to the guidelines of the declaration of Helsinki. A written informed consent was obtained from the participants and investigations were approved by the ethics committees of the Universities of Düsseldorf, Essen, Münster, Stellenbosch and Cape Town. All German subjects were male. Both SA uninfected and HIV-infected groups consisted of 63 % females and 37 % males. Participants had education with a high school diploma in Germany or schooling of 8 years in SA. The virus was transmitted sexually. 68 % of the German patients received antiretroviral medication (ARVs), whereas 69 % of the SA subjects were on ARVs. HIV infection in German participants was most likely acquired by homosexual transmission (study group consists of men having sex with men) and in SA by heterosexual transmission. People presenting with a neurological, psychiatric disorder, history of drug abuse or patients with opportunistic infections were excluded from this study. Candidates for HAND were screened in Germany with HDS (Power et al. 1995; Sakamoto et al. 2013) and in SA with IHDS (Sacktor et al. 2005) or GDS (Carey et al. 2004). Participants underwent neurological examination and neuropsychological testing. The neuropsychological test battery comprised tests for the following domains: attention (the Mental Alternation Test (MAT) and the Mental Control Test (MCT)), learning and memory [the Hopkins Verbal Learning Test (HVLT) and the Brief Visuospatial Memory Test (BVMT)], psychomotor speed [Finger tapping (FT) and the Grooved Pegboard Test(GP)], psychomotor speed [Trail Making Test part A (TMTA), Color TrailsTest 1(CT1) and Digit Symbol-Coding (DSC)], executive function [ColorTrails Test 2 (CT2), Stroop Colour-Word test (SCW), Wisconsin Card-Sorting Test (WCST), and language (category fluency)]. For GDS, raw test scores were converted to T scores to control for factors such as education, gender and ethnicity. Impairment was classified as having a GDS score of 0.5 or higher. This cutoff score is known to have a good positive predictor value for impairment (Carey et al. 2004).

### Routine virological and immunological analysis

HIV RNA was extracted in SA with the m2000sp Abbott Sample Preparation System (Abbott Laboratories, IL, USA) and detected with the Abbott m2000 Real-Time HIV-1 Amplification Reagent Kit. In Germany, HIV RNA was measured using Abbott RT/m2000 assay (Abbott Diagnostics, Wiesbaden, Germany). CD4 counts were determined using FACSs Calibur flow cytometers (Beckton Dickinson, Franklin Lakes, NJ, USA).

### CSF DA quantification

CSF samples for DA detection were supplemented with phosphoric acid (150 mM, Merck, Darmstadt, Germany) and stored at −80 °C. CSF DA concentration was measured using a reverse-phase high-pressure liquid chromatography system (Agilent 1100, Waldbronn, Germany) and detected by electrochemical detection (Koutsilieri et al. 1997). For qualitative and quantitative analyses, we compared retention times and peak heights with those from commercially available standards (Sigma, St. Louis, MO, USA).

### DAT genotyping

Genomic DNA of participants was purified from peripheral blood mononuclear cells (PBMC) with the QIAamp DNA Mini Kit (Qiagen). PBMC were isolated using Histopaque-1077 gradient centrifugation (Sigma-Aldrich, St. Louis, MO, USA). The DAT1 3′ variable number tandem repeat (VNTR) polymorphism of SLC6A3 was analyzed with *Taq*-polymerase chain reaction (PCR) (New England Biolabs, Ipswich, MA, USA) using the forward and reverse primers TGTAGGGAACGGCCTGAGAG and CTTCCTGGAGGTCACGGCTCAAAGG (Vandenbergh et al. 1992). PCR products were subjected to 3 % agarose gels which contained ethidium bromide and analyzed for the amplified fragment length polymorphisms by comparison with molecular weight standards.

### DAT mRNA expression

RNA of SA patients was isolated from PBMC using the RNeasy Plus Mini Kit (Qiagen, Hilden, Germany). After reverse transcription of RNA with the iScript cDNA Synthesis Kit (Bio-Rad, Hercules, CA, USA), cDNA was analyzed by quantitative real-time PCR (qPCR) using DyNAmo ColorFlash SYBR Green qPCR Kit (Finnzymes, Espoo, Finland). The qPCRs were run in duplicates in the Bio-Rad iCycler (Bio-Rad) using the primers DAT fwd (5′-TCCTGGAACAGCCCCAACT-3′) and DAT rev (5′-TGTGGTCCCAAAAGTGTCGTT-3′) (Mill et al. 2002). Amplification was carried out in a final volume of 20 μl containing 1 μl cDNA, 0.5 μM primer and 0.01 μM fluoresceine (Flouresceine Calibrator Dye, Bio-Rad) with 45 cycles of 10 s at 95 °C and 5 s 67 °C. Gene expression levels were calculated as relative expression compared to the housekeeping gene β-actin (QuantiTect Primer assay, Qiagen) using a standard curve and analyzed with the Bio-Rad iCycler iQ system program (copies hDAT/copies β-actin).

### Statistical analysis

To assess the influence of the DAT genotype on HIV infection risk, an unconditional direct logistic regression model was performed where the odds ratio and 95 % confidence interval (CI) were computed with an adjustment for the two potential cofounders’ gender and ethnicity. Due to the small number of potential cofounders available and without having an a priori order of importance of those, the direct regression was preferred over a sequential type. Statistical differences in genotype and allele frequencies of the subgroups were analyzed using the Fisher’s exact test. To compare the means of two groups, we used the unpaired *t* test if values were normally distributed and the Mann–Whitney *U* test for non-normally distributed values. All statistical tests were performed with the Software Package for Social Sciences (SPSS) version 20 and Prism 6 for Mac OS X. All *p* values were two-sided, a *p* value of 0.05 or less was considered statistically significant.

## Results

### Patients

German study participants had a mean age of 46.7 ± 10.3 years and SA 36.7 ± 9.3 years (Table 1). HIV patients in both study groups presented with similar CD4 counts. Plasma viral load in therapy-naïve patients was similar between German and SA participants, whereas SA patients treated with ARVs exhibited a 4.5-fold higher viral load than those in German patients, suggesting worse treatment adherence in SA (Table 1). CSF viral load of the German participants on ARVs was 76-fold less compared to therapy-naïve patients. 32 % of the German and 23 % of the SA patients were diagnosed as candidates for cognitive impairment according to the HDS for German and IHDS or GDS for SA subjects.

**Table 1:** Clinical characteristics of HIV patients

	German study participants			South African study participants		
	All subjects	w/o HAART	HAART	All subjects	w/o HAART	HAART
Patients	72	23	49	54	17	37
Age (years)	46.7 ± 10.3 (23.2–70.9)	44.1 ± 11.9 (23.2–68.0)	47.9 ± 9.4 (29.0–70.9)	36.7 ± 9.3 (24–75)	37.5 ± 12.8 (25–75)	36.5 ± 7.8 (24–54)
Duration of HIV diagnosis (years)	7.1 ± 5.7 (0.02–20.2)	3.3 ± 3.5 (0.02–11.9)	8.9 ± 5.6 (0.22–20.2)	3.8 ± 2.2 (0.8–8)	3.1 ± 1.7 (0.8–4.8)	3.9 ± 2.3 (0.8–8)
CD4+ cells (cells/μl)	457.4 ± 292.5 (9–1,400)	488.1 ± 315 (17–1,400)	443.0 ± 283.6 (9–1,091)	427.8 ± 263.4 (86–1,436)	392.9 ± 175.8 (86–741)	447.0 ± 302.3 (86–1,436)
Plasma viral load (copies/ml)	17,374 ± 53,993 (1–313,562)	35,649 ± 69,995 (87–313,562)	9,169 ± 43,431 (1–295,833)	39,585 ± 128,650 (1–800,000)	37,060 ± 43,386 (40–143,439)	40,945 ± 157,591 (1–800,000)
CSF viral load (copies/ml)	9,604 ± 59,619 (1–500,000)	29,244 ± 104,277 (1–500,000)	385 ± 1,505 (1–8,076)	n.d.	n.d.	n.d.
HDS/IHDS	12.28 ± 4.03 (3.5–16)	11.52 ± 4.11 (5–16)	12.64 ± 3.99 (3.5–16)	9.39 ± 1.81 (7–12)	n.d.	9.39 ± 1.81 (7–12)
GDS	n.d.	n.d.	n.d.	0.21 ± 0.39 (0–1.64)	0.24 ± 0.31 (0–1.08)	0.19 ± 0.46 (0–1.64)

Values are mean ± SD. Numbers in parenthesis show range of values. No statistical significance was detected (Mann–Whitney *U* test for non-parametric distributed values or unpaired *t* test)

*HDS* HIV dementia score, *IHDS* international HIV dementia score, *GDS* global deficit score, *n.d.* not determined

### Frequency of DAT alleles

**Fig. 1:**
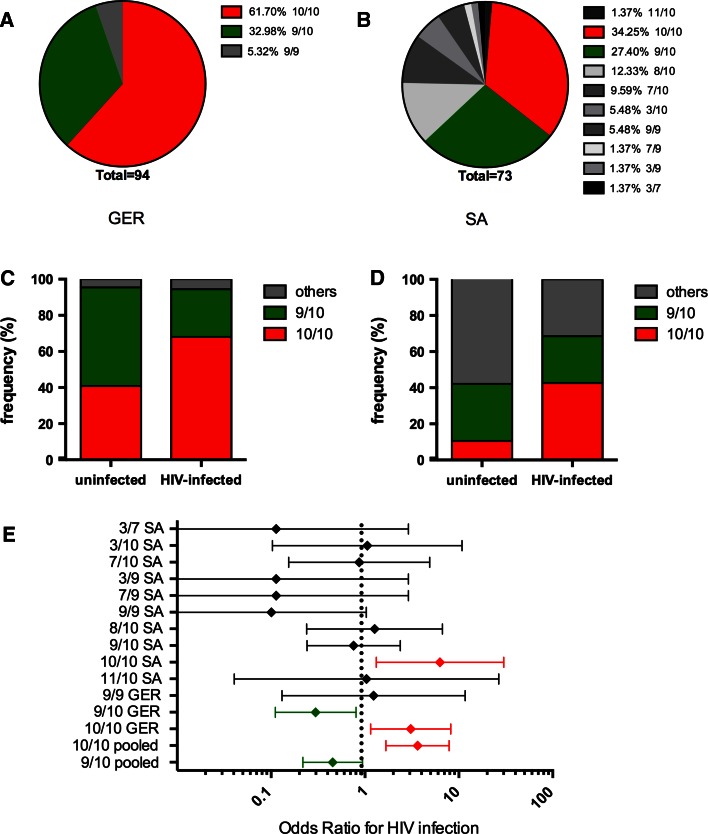
Distribution of the DAT polymorphism in German (GER) and South African (SA) populations and its association with the HIV infection. **a** Distribution of different DAT polymorphisms in Germany with 94 individuals. **b** Distribution of different DAT polymorphisms in SA with 73 individuals. **c** DAT alleles in uninfected (*n* = 22) and HIV-infected (*n* = 72) study participants in Germany (*p* = 0.0266, Fisher’s exact test). **d** DAT alleles in uninfected (*n* = 19) and HIV-infected (*n* = 54) study participants in SA (*p* = 0.0121, Fisher’s exact test). **e** Risk of acquiring HIV infection associated with the different DAT genotypes. Odds ratios for HIV infection are shown in diamonds ±95 % confidence intervals. Polymorphisms associated with an increased infection risk are shown in *red*, polymorphisms associated with a reduced infection risk are shown in *green*. In addition to a separate analysis of the odds ratios for each study group, the 10/10 and 9/10-repeat alleles are also depicted as pooled data from the two study groups

The two ethnic different cohorts exhibited a diverse allele distribution in the examined DAT polymorphism (Fig. 1a, b). In Germany, three genotypes were detected including the 10/10- (62 %), the 9/10- (33 %) and the 9/9-repeat allele (5 %) (Fig. 1a). This distribution is in accordance with previous studies in European populations (Kang et al. 1999). In SA, a greater allele diversity was observed including 3/7, 3/9, 3/10, 7/9, 9/9, 7/10, 8/10, 9/10, 10/10, and 10/11 genotypes with the 10/10- and 9/10-repeat alleles being the most frequent (Fig. 1b).

### Correlation of DAT genotypes with HIV infection

Both in Germany and SA, the DAT 10/10-repeat allele was found at significantly higher frequency in HIV-infected patients than in uninfected individuals (Fig. 1c, d). 68.1 % of the HIV-infected patients and 40.9 % of the uninfected carried the 10/10 allele in Germany. In SA, 42.6 % HIV-infected and only 10.5 % uninfected carried this allele (*p* = 0.0266 for Germany and *p* = 0.0121 for SA, Fisher’s exact test) (Fig. 1c, d). The odds ratio for HIV infection associated with the DAT 10/10 genotype was 3.08 (95 % CI 1.15–8.23) for the Germans and 6.31 (95 % CI 1.32–30.06) for SA (Fig. 1e). Logistic regression analysis adjusted for gender and ethnicity in pooled cohorts showed and odds ratio of 3.93 (95 % CI 1.72–8.96) for the DAT 10/10 allele (*p* = 0.001), indicating a substantially higher risk for HIV infection if the DAT 10/10 genotype is present (Fig. 1e).

### Functional changes associated with the DAT 10/10 genotype

**Fig. 2:**
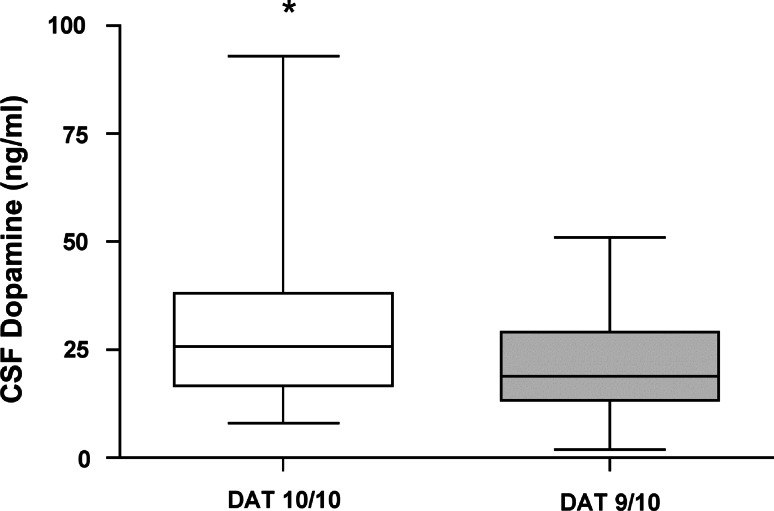
Participants carrying the DAT 10/10-repeat allele have higher levels of CSF DA compared with those with 9/10-repeat allele. DA concentration in CSF of German participants with 10/10-repeat allele (*n* = 55) and 9/10-repeat allele (*n* = 28). *Box plots* show the medians with upper and lower quartiles. *p = 0.03, significantly different from 9/10-repeat allele carriers (Mann–Whitney *U* test for non-parametric distributed values)

**Fig. 3:**
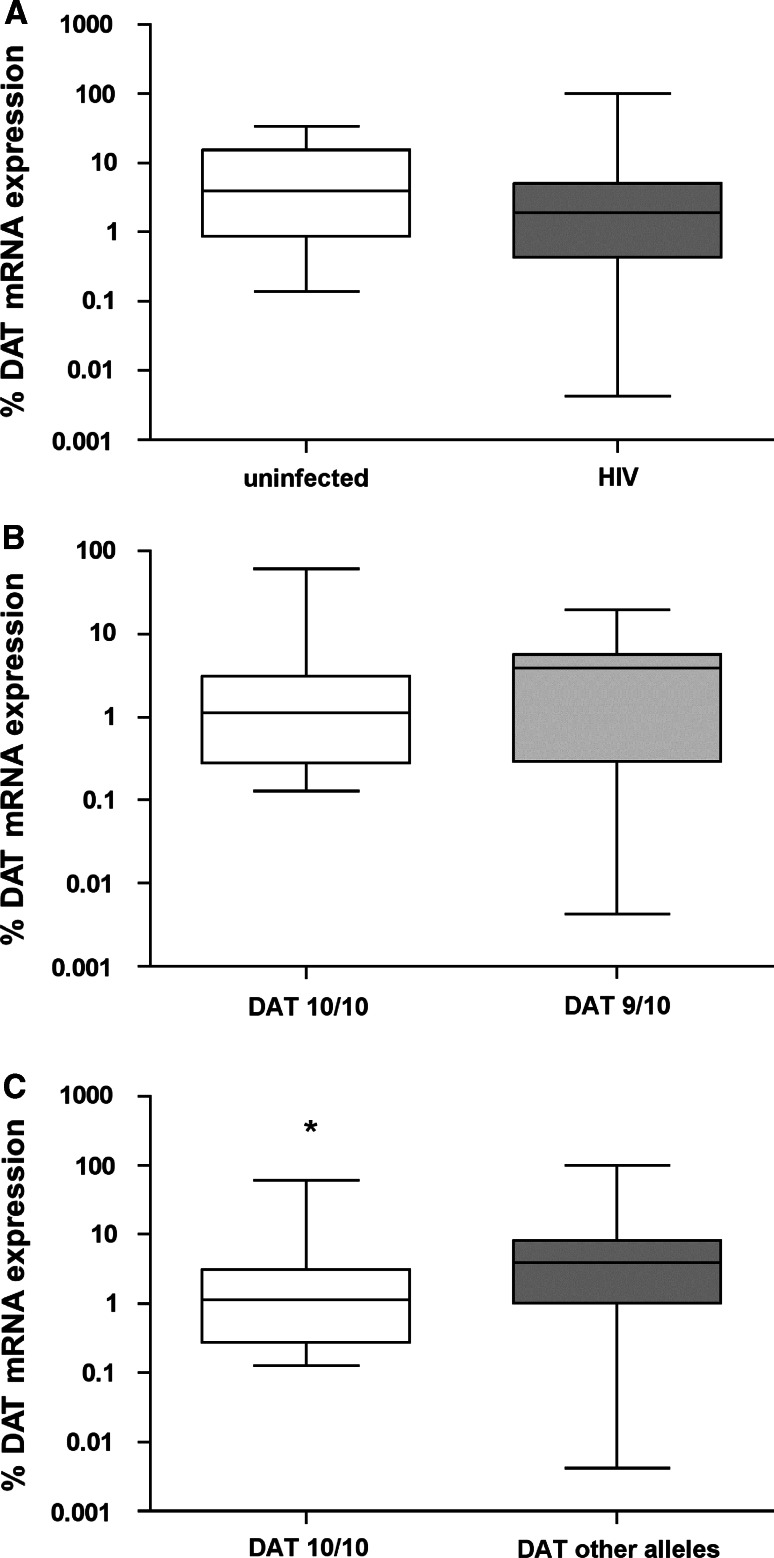
Participants carrying the DAT 10/10-repeat allele have reduced levels of PBMC DAT mRNA expression compared with those with other DAT alleles. **a** DAT mRNA expression in PBMC of SA uninfected (*n* = 17) and HIV-infected subjects (*n* = 37); **b** DAT mRNA expression in PBMC of SA with DAT 10/10 (*n* = 17) and 9/10-repeat allele (*n* = 14); **c** DAT mRNA expression in PBMC of SA with DAT 10/10 (*n* = 17) compared with other DAT alleles (3/7, 3/9, 3/10, 7/9, 9/9, 7/10, 8/10, 9/10 and 10/11; *n* = 37). *p = 0.05, significantly different from DAT other allele carriers (Mann–Whitney *U* test for non-parametric distributed values)

Subjects homozygous for the DAT 10-repeat allele exhibited a significantly higher DA availability compared to carriers of the second most frequent 9/10-repeat allele (Fig. 2). CSF DA was measured only from German subjects because CSF from SA was not available. To compensate for this lack and to gain information on the DA availability in the periphery, we measured the expression of DAT mRNA in PBMC of SA participants. DAT expression was similar between HIV-infected and uninfected subjects (Fig. 3a) and DAT mRNA did not differ between people carrying the 10/10- and 9/10-repeat alleles (Fig. 3b). However, we found a significantly lower mRNA DAT expression in these subjects who were homozygous for the 10-repeat allele in contrast to all other genotypes (Fig. 3c).

No relation between DAT genotype and disease progression markers such as CD4 count and viral load was evident (Table 2). Carriers of the DAT 10/10-repeat allele showed a trend towards higher frequency of neurocognitive impairment (32 %) according to the HDS/IHDS and GDS compared to 21 % impairment for carriers of the non-10/10 alleles (Table 2). However, this was not statistically significant due to the fact that our study was not powered for disease severity and this n was not large enough to answer this question.

**Table 2:** Influence of DAT 10/10-repeat allele compared to other DAT alleles on HIV disease severity

DAT alleles	10/10 (*n* = 68)	9/10 (*n* = 27)	DAT other than 10/10 (*n* = 47)
CD4+ cells (cells/μl)	443.2 ± 297.9	443.0 ± 298.0	449.6 ± 256.1
HIV RNA (copies/ml blood)	24,747 ± 60,045	12,285 ± 28,229	27,397 ± 119,995
Neurocognitive impairment (%)	32.3 %	20 %	21.7 %

Data are mean ± SD. No statistical significance was detected (Mann–Whitney *U* test for non-parametric distributed values and Fisher’s exact test)

## Discussion

In this study, we demonstrate for the first time that people infected with HIV carry significantly more often the DAT 10/10-repeat allele compared to uninfected subjects, indicating that this genotype confers a significant risk factor for HIV infection. The reason for studying both German and SA groups was that we initially identified the higher frequency of the DAT 10/10-repeat allele in our German cohort and we wanted to exclude that this could be a feature based on homosexuality as the German participants are men having sex with men. This is why we chose to investigate further this phenomenon in a completely different setting, with heterosexuals with different ethnicity, gender and even virus subtype. Indeed our finding on the frequency of DAT 10/10-repeat allele in HIV-infected subjects is independent on ethnicity, gender, route of sexual transmission and viral subtype (HIV infections in SA are predominantly attributed to subtype C (Jacobs et al. 2009) and in Germany to subtype B (Paraskevis et al. 2009). The independent detection in these different study populations emphasizes the importance of our findings, even though the number of screened patients is comparably low.

One possible explanation for the higher frequency of the 10/10-repeat allele in HIV patients could be that people carrying this allele have a different behavior including a risky, impulsive way of life. In this line, the DAT 10/10-repeat allele has been associated with the attention deficit hyperactivity disorder (ADHD) (Hawi et al. 2005), a disease with enhanced impulsivity (Cornish et al. 2005). Individuals with the highest hyperactivity and impulsivity scores were found to be homozygous for the DAT 10-repeat allele along with one 7-repeat variant for DRD4 (Roman et al. 2001). The same genetic variants were associated with reduced inhibition control (Congdon et al. 2008). Interestingly, people with the 10/10-repeat allele showed more risk taking in a risk-taking task (Mata et al. 2012). In studies with humans, the DAT 10/10-repeat allele has been associated with changes in DAT availability, however, without consistent results. Individuals with DAT 10-repeat allele have lower (van de Giessen et al. 2009) or higher DAT availability (Heinz et al. 2000) than those with other DAT genotypes. The main role of DAT is to regulate DA availability (Giros and Caron 1993) by forward and reverse transport mechanisms that result in changes of synaptic DA in an opposite mode (Uhl 2003). To check whether concentrations of DA in HIV-infected subjects were attributed to the DAT genotype, we measured concentrations of DA in CSF which is a useful index of central DA activity in humans, particularly for the striatum (Wester et al. 1990). We found that participants with the 10/10-repeat allele had higher levels of CSF DA. This is in accordance with the only study in the literature to our knowledge, which reported on DAT genotypes and CSF DA (Wagner et al. 2007). Those authors, like us here, reported that individuals with the DAT 10/10-repeat allele have higher CSF DA levels compared to DAT 9/10 and DAT 9/9 genotypes in traumatic brain injury patients (Wagner et al. 2007).

We also found that 10/10-repeat allele carriers had a significantly lower PBMC DAT expression compared to all other genotypes. This allows us to assume indirectly on the alterations in DA availability in the SA participants because the peripheral PBMC DAT correlates significantly with the central striatal DAT expression in people with dopaminergic medication (Pontieri and Colosimo 2010). In accordance different genotypes of the DAT1 3′ VNTR influence mRNA expression of the DAT both in brain tissue and lymphocytes, although the expression of DAT in the periphery is approximately 1,000-fold lower than in the brain (Mill et al. 2002).

If the DAT 10/10-repeat allele leads to an elevated DA availability, as we show here, it would be possible that it causes an exacerbation of CNS HIV infection, as it was demonstrated previously that SIV neuropathology was accelerated due to dopaminergic drugs (Czub et al. 2001). This issue could not be approached in living people but neurocognitive scores did not seem to alter in DAT 10/10 carriers with HIV infection. In another study, it was shown that the dopaminergic drug selegiline, known to increase DAT expression (Lamensdorf et al. 1999), was well tolerated by HIV-infected patients with cognitive impairment based on the neuropsychology scores (Evans et al. 2007; Schifitto et al. 2007). Another discussion point is that an increase of DA in the periphery by the DAT 10/10-repeat genotype may result in an increased infection risk due to the effects of DA on the immune system per se. It is known that DA may regulate the initiation and development of immune responses (Buttarelli et al. 2011). It has been reported that stimulation of DRD1 receptors, of which the most prominent agonist is DA itself, inhibits the cytotoxic function of CD8+ T cells (Saha et al. 2001), which play a crucial role in the immune response towards infections. Individuals with higher DA levels may, therefore, be more prone towards sexually-transmitted diseases such as herpes genitalis or syphilis, which correlate with a 2–5-fold higher infection risk towards HIV. Besides this indirect effect of DA, DA may also directly enhance HIV replication. We have shown previously that DA activates HIV replication in latently infected T cells in vitro (Scheller et al. 2000), so that individuals with higher DA levels might have an elevated infection risk towards HIV due to a faster initial virus replication. As we do not have information on the virological and immunological status of the HIV patients in the beginning of their infection, we cannot know whether the participants with 10/10-repeat allele had a different initial disease progression. In addition, as our study was not powered for disease severity, we can only assume from our results that later on the DAT 10/10-repeat allele may have no effect on CD4+ T cells, viral load and neurocognitive impairment. This is in agreement with a previous study which found no effect of DAT genotype on disease severity and brain function in HIV-infected patients (Levine et al. 2012) while another group identified that the DRD3 genetic polymorphism relates with cognitive impairment in methamphetamine/HIV-infected people (Gupta et al. 2011).

Taken together, this is the first study to show that increase in CSF DA levels in HIV-infected subjects is attributed to the DAT genotype and it is not necessarily a secondary effect of the virus. In addition, HIV-infected individuals carry more frequently a genetic variant in a functional element of the dopaminergic synapse such as DAT. This could be due to a behavioral trait with a severe consequence but we cannot rule out the possibility that the population with HIV has another significant risk factor that has enriched the frequency of this repeat in these patients. The strengths of our study are the high odds ratios in both German and SA groups, as well as the fact that the results were similar in different ethnicities, were independent on gender, routes of sexual transmission and viral subtype. The weakness of the study is that the study sample with 167 participants was modest. However, the sample size provided enough power to detect statistical significance between frequency of HIV infection and the DAT variant. New studies have to be planned with a large sample number to confirm our data and experimental studies have to be designed to further elucidate the mechanistic background of these data.
